# Parsonage-Turner syndrome: a long-term follow-up study of 42-cases in Guangxi, China

**DOI:** 10.3389/fneur.2026.1804804

**Published:** 2026-04-07

**Authors:** Guang-Yao Li, Chang-Tai Luo, Yi-Jin Gao, Jia-Hui Wang, Ke Sha

**Affiliations:** 1 Department of Orthopedic Trauma and Hand Surgery, The First Affiliated Hospital of Guangxi Medical University, Nanning, Guangxi, China; 2 Affiliated Hospital of Youjiang Medical University for Nationalities, Key Laboratory of Clinical Cohort Research on Bone and Joint Degenerative Diseases of Guangxi, Baise, Guangxi, China; 3 Department of Orthopedics, Wuming Hospital of Guangxi Medical University, Nanning, Guangxi, China

**Keywords:** clinical outcome, diagnosis, epidemiology, Parsonage-Turner syndrome, treatment

## Abstract

**Introduction:**

Parsonage-Turner syndrome (PTS) is a rare peripheral neuropathy with variable clinical manifestations. Its true incidence is higher than previously recognized, characterized by a prolonged recovery period, potential residual disability and limb pain, which poses a persistent challenge for clinic.

**Methods:**

Forty-seven patients with PTS were admitted to our hospital’s hand surgery center between November 2017 and November 2022, 42 of whom were included in the retrospective study, with the exception of 5 patients whose data were incomplete. Demographic information, clinical data, and auxiliary examination results were collected, and all patients were regularly followed up. Outcomes were evaluated via the Medical Research Council (MRC) muscle strength grading scale and visual analogue scale (VAS) pain score. SPSS 22.0 software was used for statistical analysis.

**Results:**

The annual incidence of PTS was 47 per 100,000 individuals. The 42 patients, 27 males and 15 females, had an average age of 41.1 ± 20.3 years. The average period between onset and diagnosis was 60 days, and the right or dominant limb was more commonly affected. The most frequent initial symptom was spontaneous pain (69%), with the majority of cases affecting the shoulder girdle (72%). The suprascapular nerve (52%), long thoracic nerve (50%), and axillary nerve (38%) were the most frequently affected nerves. Seven individuals underwent surgery because of failure to respond or poor recovery after at least 3 months of nonsurgical treatment. The average follow-up time was 54.8 ± 15.0 months. Most patients achieved effective recovery within two years, with 18 attaining complete recovery and 13 achieving partial recovery (MRC Grade 4). Twenty-one patients experienced chronic pain.

**Conclusion:**

The frequency of PTS in hand surgery center is relatively high, and the epidemiological patterns are similar to those reported in previous studies. However, the majority of patients experience untimely treatment, which results in residual pain and inadequate motor function recovery. Enhancing clinical awareness of PTS for early diagnosis should be prioritized, particularly in cases of suprascapular nerve dysfunction combined with long thoracic nerve dysfunction or isolated posterior interosseous nerve dysfunction following spontaneous pain.

## 1 Introduction

Parsonage-Turner syndrome (PTS), also known as neuralgic amyotrophy or brachial plexitis, is a disabling peripheral neuropathy characterized by acute pain as the initial symptom. The pain is typically spontaneous, severe, and meets the criteria for neuropathic pain (1). In the absence of trauma, malignant tumors or a history of radiotherapy, the brachial plexus (BP) is the primary target of PTS, although the cervical and lumbosacral plexuses may also be involved (1). Currently, PTS is presumed to be associated with factors such as viral infection, immunization, aberrant autoimmune activation, and genetic predisposition, while the specific mechanism is still unclear (2). PTS was previously considered a rare disease, with an annual incidence of 2**–**3 per 100,000 (2). However, recent research has revised this view: Milner et al. (3) reported an annual incidence of 34 per 100,000 in an American hand surgery center, and van Alfen et al. (4) demonstrated that after general practitioners received specialized training in PTS diagnosis and treatment, the annual incidence reported by primary healthcare institutions in the Netherlands reached as high as 1 per 1,000 people. In practice, most doctors lack a thorough grasp of PTS, which leads to missed diagnoses, misdiagnoses, and delayed treatments, potentially leading to a higher real incidence rate. Therefore, diagnosing PTS remains a significant difficulty in orthopedics, neurology, and neurosurgery.

PTS typically progresses in three stages: pain, paralysis/sensory loss, and sluggish recovery. Asymmetric localized paralysis, muscular atrophy, and sensory abnormalities are the primary clinical signs after the pain stage (5). In the past, there was a belief that 80 to 90% of patients with PTS could fully recover on their own (6, 7). However, recent studies have indicated that recovery can take up to several years, with approximately one-third of PTS patients experiencing chronic pain, more than half having persistent dysfunction, and some even experiencing long-term disability, which significantly disrupts their life (3, 8–10). It is apparent that the prognosis of PTS is often poor, requiring more attention. Moreover, long-term follow-up studies on PTS prognosis in Asian populations are lacking, constituting a research gap. Therefore, this study retrospectively analyzed the clinical data of PTS patients treated at our center, systematically characterized their epidemiological features, and performed follow-up surveys. The goal was to provide evidence-based support for improving diagnosis and treatment efficiency, as well as optimizing PTS patient prognosis.

## 2 Methods

### 2.1 Study object

This study (No. 2016-E0024) was approved by the Ethics Committee of The First Affiliated Hospital of Guangxi Medical University. A total of 47 PTS patients were treated at this medical center between November 2017 and November 2022. Every patient met the diagnostic standards proposed by van Alfen et al. (4): (1) acute or subacute onset; (2) initial pain score > 7/10; (3) multifocal neuropathy predominantly involving the upper BP (with or without winged scapula); (4) monophasic course sequentially progressing through pain stage, functional deficit stage, and recovery stage; (5) no history of local trauma, malignant tumors, or radiotherapy; and (6) completion of electrodiagnostic (EDX) examinations; some patients also underwent MRI or ultrasound based on clinical indications. Of the 47 patients initially assessed, 5 were excluded due to incomplete data, leaving a final cohort of 42 (89.4%).

### 2.2 Patient data

The collected data included: (1) demographic data: gender, age, body mass index (BMI), and occupation; (2) medical history: onset time, prodromal symptoms or precipitating factors, number of episodes, treatment course, past history (including hepatitis virus infection), and family history; (3) clinical data: affected side and dominant limb, pain location and duration, and systemic physical examination. Tendon reflexes and pathological signs were assessed to exclude central nervous system lesions. Muscle strength, sensory function, and muscle tone were thoroughly evaluated. Muscle weakness was graded using the Medical Research Council (MRC) scale (0–5), and the findings were correlated with the specific motor neurons innervating the affected muscle groups. Sensory function was assessed using standardized clinical tests, including light touch (with cotton wisp), pinprick (with disposable safety pin). Sensation was graded as normal, impaired (hypoesthesia), or absent. The dermatomal distribution of sensory deficits was mapped to the affected nerves. Limb pain was subjectively assessed using the visual analogue scale (VAS). (4) auxiliary examinations: electromyography (EMG), magnetic resonance imaging (MRI), and ultrasound results were obtained. EMG revealed axonal neuropathy involving one or more nerves, nerve fascicles, nerve roots, or segments of nerve trunks, including branches of the BP (11). MRI clearly demonstrated the anatomical relationship between the lesions and adjacent tissues, whereas ultrasound enabled continuous real-time assessment of BP morphology and course at multiple body positions (12, 13).

### 2.3 Clinical treatment

Initially, nonsurgical treatment was administered to all patients according to clinical stage and symptoms. This comprised neurotrophic therapy, pain control, and early immunomodulatory treatment: corticosteroids or intravenous immunoglobulin are strongly recommended within two weeks of onset (1, 3, 14). Rehabilitation treatment was the core strategy, with appropriate immobilization and protective measures. Patients who failed to respond to nonsurgical treatment or demonstrated poor recovery—defined as insignificant improvement in muscle strength (MRC grade <3) after 3–6 months of comprehensive nonsurgical management—underwent surgical evaluation (1). Surgical approaches, including neurolysis, nerve transplantation, nerve transfer, or tendon transfer, were chosen based on the specific conditions of each patient.

### 2.4 Follow-up survey and data analysis

All patients were followed up monthly for the first 3 months post-treatment, then every 3 months thereafter at outpatient clinics. According to the criteria proposed by van Alfen (9, 15), functional recovery was categorized into four groups: complete recovery without residual pain (MRC grade 5); complete recovery with residual pain (MRC grade 5); moderate recovery (MRC grade 4); and poor recovery (<MRC grade 4). Furthermore, patients were stratified into four groups based on the time to recovery: Group I (0–6 months), Group II (6 months–1 year), Group III (1–2 years), and Group IV (> 2 years). Data were analyzed using SPSS version 22.0 (IBM Corp., Armonk, NY, USA). Categorical data are presented as percentages, and continuous data are described as mean ± SD for normally distributed data or as medians for skewed data.

## 3 Results

### 3.1 Epidemiology

During the 6-year study period (2017–2022), 47 PTS patients were identified among 100,000 patients treated at our center. The annual incidence was 47 per 100,000, 15.7 times higher than the incidence in the general population. Among the 42 patients included in this study, 44 upper limbs were affected. The right and dominant upper limbs were affected more frequently, accounting for 66% and 68%, respectively (Table 1). The male-to-female ratio was 1.8:1, with 27 males and 15 females. The mean age was 41.1 ± 20.3 years (range: 3 months–74 years), and patients aged 40–49 years comprised the largest proportion (29%). Surprisingly, the average time from onset to diagnosis was 60 days. Precipitating conditions were identified in 19 patients (45%), including vaccination (21%), viral infection (19%), and immunosuppressive therapy (5%). Additionally, one patient (2.4%) experienced recurrence, and no hereditary cases were identified.

**Table 1:** Epidemiological data of the 42 patients.

Item	Mean ± SD
Age (year)	41.1 ± 20.3
Gender (male)	27 (64%)
BMI (kg/m^2^)	21.4 ± 2.5
Blue-collar workers	12 (29%)
Precipitating conditions	19 (45%)
Right side involved	29 (66%)
Dominant side involved	30 (68%)
Recurrent cases	1
Hereditary cases	0

### 3.2 Clinical manifestations

Sixty-nine percent of patients experienced spontaneous pain as the initial symptom, with an NRS score ≥ 7/10, and 57% reported prominent nocturnal pain (Table 2). Pain predominantly involved the shoulder girdle (72%), with a mean duration of 14 days, while muscle weakness developed on average within 3 days of onset, with the highest proportion (30%) occurring within 24 h. Analysis of nerve involvement revealed that the suprascapular nerve (SSN) (52%), long thoracic nerve (LTN) (50%), and axillary nerve (38%) were the three most commonly afflicted nerves. Multiple nerve involvement occurred in 29 patients (69%). The combination of SS*N* + LT*N* + axillary nerve was the most common (34%), followed by SS*N* + LTN (21%). Furthermore, the posterior interosseous nerve (PIN) (38%) was the most frequently involved in patients with single nerve involvement. The detailed distribution of nerve involvement is shown in Table 3. All nerve involvement data were derived from nerve conduction studies (NCSs) and needle EMG results. Typical NCS findings include decreased compound muscle action potential (CMAP) and sensory nerve action potential (SNAP) amplitudes, which are consistent with axonal injury. Needle EMG showed denervation characteristics in the target muscles, including fibrillation potentials and positive sharp waves. Several weeks after paralysis onset, needle EMG often revealed polyphasic motor units with spontaneous contraction, indicating nerve regeneration. Some patients showed decreased motor unit recruitment without denervation changes, suggesting a partial BP conduction block.

**Table 2:** The information about pain at the onset of a Parsonage-Turner syndrome.

Item		No. of patients
Precipitating conditions	Vaccination history	9 (21.43%)
	Respiratory tract infection	8 (19.05%)
	Immunosuppressor	2 (4.76%)
	No cause	23 (54.76%)
Pain before muscle weakness	Yes	29 (69.05%)
	No	13 (30.95%)
Nighttime pain	Yes	24 (57.14%)
	No	18 (42.86%)

**Table 3:** List and frequency of the different nerve lesions found during PTS.

Nerve/muscle	Parsonage and Turner (35)(*n* = 136)	Turner and Parsonage(36)(*n* = 82)	Cruz Martinez et al., (37)(*n* = 40)	Van Alfen and van Engelen (9)(*n* = 246)	Ferrante and Wilbourn (24)(*n* = 281)	Seror (16)(*n* = 355)	Li (2026)(*n*=42)
Long thoracic/serratus anterior	7%	48%	18%	70%	42%	38%	50%
Suprascapularis/supra- and infraspinatus	7%	34.15%	63%	72%	58%	37%	52%
Spinal accessory/trapezius, SCM	7%	?	?	20%	4%	14%	10%
Axillary/deltoid	3%	43%	53%	46%	25%	8%	38%
Musculocutaneous nerve/biceps	4%	16%	28%	61%	15%	?	24%
Anterior interosseous nerve (AIN)	6%	10%	13%	33%	29%	16%	10%
Posterior interosseous nerve (PIN)	4%	10%	3%	?	3%	4%	12%
Complete						8%	5%
Radial sensory	0%	0%	0%	?	0%	2%	2%
Lateral antebrachial cutaneous nerve (LABCN)	0%	0%	3%	?	5%	12%	7%
Phrenic/diaphragm	0%		3%	6.6–14%	6%	2%	2%
Lumbo-sacral plexus	0%			8.2–32%		3%	0%
Multiple mononeuropathies	42%	?	75%	?	54%	26%	69%
Bilateral lesions	29%	?	18%	28%	18%	6%	5%
Recurrent lesions	?	8%	17%	INA 26%	15%	3%	2%
				HNA 74%			
Hereditary cases	0%	0%	0%	19%		0%	0%

The frequencies are presented in decreasing order, and are issue from 6 previously published series of the literature and from the present series.

### 3.3 Follow-up outcomes

**Figure 1:**
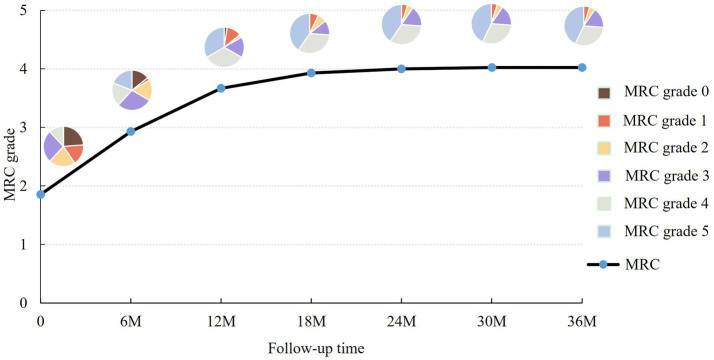
The muscle strength of PTS patients fluctuated during the 36-month follow-up. The black line represents the average MRC grade of PTS patients at different follow-up time points (6 M = 6 months, 12 M = 12 months, 18 M = 18 months, 24 M = 24 months, 30 M = 30 months, 36 M = 36 months). The pie charts at each time point show the distribution of patients according to MRC grades. The average muscle strength improved rapidly in the early stage (0–12 M) and stabilized at MRC grade 4 after 18 months of follow-up.

All patients were followed up for more than 3 years, with an average time of 54.8 ± 15.0 months. Seven patients underwent surgical intervention due to poor recovery during the follow-up period, including 2 cases of neurolysis, 2 cases of nerve transplantation, 1 case of nerve transposition, and 2 cases of tendon transfer. The surgery did not result in any PTS recurrence. At the final follow-up, 18 patients had fully recovered muscle strength (MRC grade 5), 13 patients had partially recovered (MRC grade 4), and 11 patients had poor recovery (<MRC grade 4) (Table 4). Stratified by recovery time, 8 patients recovered within 6 months, 6 within 6–12 months, 3 within 1–2 years, and 1 after more than 2 years (Table 5). The trend of muscle strength change during three years is shown in Figure 1. Notably, 21 patients (50%) experienced residual pain that impaired daily life.

**Table 4:** Patient classification into 1 of 4 groups based on their level of recovery in accordance with the grading system used by Van Alfen et al. (10)

Level of recovery	No. of cases	Percentage (%)
Complete recovery with no residual pain (Power 5/5)	10	24%
Complete recovery with some residual pain (Power 5/5)	8	19%
Partial recovery with or without residual pain (Power 4/5)	6	14%
Poor recovery with or without residual pain (Power ≤ 3/5)	5	12%

**Table 5:** Delay to full recovery broken down into 4 groups based on timescale.

Group	No. of months to recovery	No. of patients
I	0–6 months	8
II	6–12 months	6
III	1–2 years	3
IV	More than 2 years	1

## 4 Discussion

### 4.1 Epidemiology

The epidemiological characteristics observed in this study are similar to those reported in most previous studies, particularly in terms of age and sex distributions. Seror (16) reported 218 males and 137 females among 355 PTS patients, with a mean age of 42.7 ± 15.3 years (range from 12 to 87 years). A study of 246 PTS patients by van Alfen & van Engelen showed that females accounted for 32% and males 68%, with a mean age of 41.3 years (9). However, the average BMI in this study was lower than that in other studies (3, 9, 16), which may be attributed to the differences in body size between Asian and European/American populations.

Notably, the incidence of PTS in this study (47 per 100,000 people) was higher than that reported in most previous studies (3, 9, 16). Possible explanations are as follows: (1) the large regional population base and our medical center’s role as a referral center for complex cases; (2) the COVID-19 pandemic fell during the study period, which may have exposed more vulnerable people and caused more PTS due to widespread viral infections and mass immunizations (5); and (3) the physicians at our research center have received specialized training in PTS, leading to higher diagnostic accuracy. This conclusion is also supported by other studies, which reported that owing to the widespread underdiagnosis and misdiagnosis of PTS, the true annual incidence of PTS may exceed 20–30 per 100,000 people, particularly in orthopedic outpatients (3, 4).

PTS was more common in the right or dominant limb (66%). Previously, this phenomenon was thought to be associated with repeated microtrauma to the cervical nerves in right-handed individuals (16). However, blue-collar workers—who have higher intensity and frequency of dominant upper limb activity—accounted for only 29% in our cohort, which seems contradictory to previous viewpoint. On the other hand, PTS has a certain capacity for self-healing, and some patients who have mild symptoms in the nondominant limb may not seek treatment since they do not exhibit obvious disability, potentially leading to statistical bias.

The currently recognized causes of PTS include viral infection and immune factors. The potential mechanism may be that the precursor event activates the immune response, which in turn triggers immune-mediated focal inflammation (17, 18)—a mechanism consistent with the findings of this study. Pan et al. (14) noted that hourglass-like constrictions (HGCs) in affected nerve segments are characteristic pathological changes of PTS, but the exact mechanism remains unclear. In addition, Milner et al. (3) reported that 14 of 38 PTS patients were related to surgery, although the surgical site was not anatomically related to the affected nerve. Hereditary PTS accounts for approximately 10–20% of the total number of PTS cases, and is related to SEPT9 gene mutation (1, 19). Most cases occur in childhood and are often accompanied by special facial features, such as a narrow eye distance, small palpebral fissures, and a small mouth (1, 19, 20). However, there was only one case of PTS recurrence in this study, with no surgery-related or hereditary cases. This discrepancy may be attributed to the small sample size of this study or the heterogeneity across different populations.

### 4.2 Diagnosis

The history and clinical manifestations of PTS are diverse, so accurate medical history and detailed neurological examination are keys to diagnosis. A targeted history should be taken in suspected cases, covering recent immunizations (e.g., tetanus toxoid, influenza vaccine), viral infections (e.g., coronavirus, varicella zoster virus, dengue virus, hepatitis E virus), recent childbirth, prescribed medications (e.g., antiepileptics, immunosuppressants, antibiotics, antiretroviral drugs, botulinum toxin), and any surgical history (2, 5, 21, 22). The presence of these factors provides valuable clues for diagnosis. Furthermore, some patients may attribute their upper limb disability to a recent low-energy trauma, as they mistakenly link the two events temporally by subjective cognition. To avoid falling into the “complaint trap,” clinicians must assess the history critically by determining whether muscle weakness presented immediately after the trauma or developed days later. On this basis, it can be determined that trauma is not the true cause. Further investigation should then focus on identifying potential precipitating factors to reconstruct the actual pathogenic process.

The limb muscle strength typically declines rapidly following the onset of spontaneous pain, which is another characteristic of PTS. Our study found that 69% of patients experienced pain before muscle weakness, and 57% had nocturnal pain. The clinical manifestations are typically linked to the pathological characteristics of PTS, which show that lymphocytes and partial neutrophils infiltrate the walls of the small blood vessels surrounding the affected nerves (14). Accordingly, we speculate that inflammation of the epineurial vessels can not only directly compromise the blood supply to nerve fibers by stimulating vascular spasm, but also indirectly induce tissue edema, leading to nerve compression. Together, these mechanisms result in acute axonal injury and induce pain. When nerve conduction function is still present, pain persists, and damaged nerve fibers gradually undergo partial or complete rupture under mechanical stress from limb movement, followed by nerve conduction dysfunction. At this time, the pain is relieved, but further obvious motor or sensory dysfunction occurs. On the other hand, the predominance of nocturnal pain may be explained by heightened pain perception in a quiet environment, as well as circadian variations in immune system activity. It is worth mentioning that the incidence of pain in pediatric patients is relatively low, which may be related to the immature immune system of children. Abnormal immune responses can occur at lower thresholds and cause neurological damage before a symptomatic pain response is mounted.

Physical examination findings are equally important. Thirty-nine percent of patients experienced muscle weakness 1 to 7 days after onset, 34% within 24 h, and muscle atrophy usually appeared between 2 and 6 weeks, followed by the recovery stage (1)—a timeline consistent with our observations. Some patients had already achieved partial functional recovery because they arrived at our center late. Therefore, a comprehensive assessment should be made in conjunction with the medical history. In particular, attention should be paid to the differential diagnosis of carpal tunnel syndrome, cubital tunnel syndrome, cervical spondylotic radiculopathy, rotator cuff injury, chronic inflammatory demyelinating polyneuropathy, vasculitis, and neoplastic brachial plexopathy (1, 23). Among the PTS patients in this group, 69% had multiple neuropathies, with high incidences of SSN (52%) and LTN (50%) lesions, which is consistent with other studies (9, 16, 24). In order to better represent the distribution of the affected nerves, the individual nerves of PTS were accurately counted, but based on our observations, we prefer to think of lesions with multiple nerve involvement are located in the BP region, rather than being affected simultaneously with multiple branches far away. Ferrante and Wilbourn (24) reported that multiple mononeuropathies accounted for 152 of 281 cases, with the most commonly involved nerves being the SSN (58%), LTN (42%), anterior interosseous nerve (AIN) (29%), and axillary nerve (25%). A systematic review by Seror (1) summarized the most frequently involved PTS muscles as the infraspinatus (72%), serratus anterior (70%), supraspinatus (65%), and biceps brachii (61%). However, the proportion of patients with axillary nerve and musculocutaneous nerve involvement in our study was higher than that reported in other studies (9, 16, 24). This may be because some studies did not further assess deltoid muscle strength when supraspinatus muscle weakness prevented shoulder abduction, leading to missed axillary nerve lesions. Musculocutaneous nerve examination details are also easily overlooked, as elbow flexion is controlled by the biceps brachii, brachialis, and anconeus muscles. Mild weakness of biceps brachii may not affect overall elbow flexion strength; however, the standard examination of the biceps (forearm supination, palm up) enables accurate evaluation of the function of the musculocutaneous nerve. Additionally, a long-term study of PTS patients in hand surgery outpatient by Milner et al. (3) revealed that the most frequently affected nerves were the axillary nerve (13%), PIN (24%), and AIN (18%), with the traditional high-incidence SSN involvement accounting for only 5%. This discrepancy may be due to most patients with shoulder dysfunction seeking treatment in orthopedic joint departments, with only a small proportion referred to hand surgery departments. This finding also suggests that PTS may present different characteristics across specialties. PTS should be seriously considered when the SSN and LTN functions are damaged simultaneously or when the PIN is impaired alone.

Auxiliary examinations should be further used to confirm PTS, particularly for atypical patients who present with muscle weakness without initial pain. These include EMG, MRI, and ultrasound. EMG, the gold standard for assessing nerve damage, varies with the underlying pathological alterations and may reveal various combinations of conduction block, demyelination, or Wallerian degeneration (1). However, active muscle denervation signals, such as fibrillation potentials, can be reliably detected only 2–3 weeks after symptom onset (25), which limits their utility for early-stage diagnosis. Ultrasound and MRI serve as first-line tools for acute-phase PTS, enabling early lesion detection and differentiation from nerve compression, tumors, and trauma. Ultrasonography offers the advantages including convenience, low cost, high sensitivity for detecting HGCs of superficial nerves (e.g., the SSN), and rapid bedside evaluation (26), MRI can identify HGCs and edema of the affected muscles, with clearer visualization of deep nerves (e.g., the axillary nerve and the LTN), providing more information to exclude structural lesions. In the late stage of PTS, muscle atrophy with fatty infiltration can also be observed on MR (27). Queler et al. (28) innovatively proposed serum neurofilament light chain (sNfL) levels as an auxiliary diagnostic indicator for early suspected cases. As a key axonal protein, sNfL is released into the bloodstream following nerve axonal damage in PTS patients within 6 months of symptom onset and remains elevated, and then gradually returns to normal. Therefore, a combination of physical examination, ancillary tests, and neurology consultation when necessary can enhance diagnostic efficiency.

### 4.3 Treatment strategy

Early and appropriate treatment results in better outcomes and minimizes the risk of sequelae. Treatment should be tailored to the specific clinical manifestations and stage of PTS. Corticosteroids or immunoglobulin provided within two weeks are the preferred option during the pain stage if a definitive diagnosis is made within one month of onset, since they may shorten the pain stage and improve prognosis (29). Unfortunately, the median time from onset to diagnosis in our cohort was 60 days, largely because many patients were referred and most clinicians failed to recognize PTS promptly, resulting in delayed early treatment. If PTS were diagnosed 1–2 months earlier, pain symptoms may be partly alleviated. At this stage, nonsteroidal anti-inflammatory drugs (NSAIDs) and/or opioids can be used to relieve symptomatic pain, combined with neurotrophic treatment. For patients diagnosed with a disease course exceeding 2 months and concurrent muscle paralysis, combined pharmacological and nonpharmacological treatments should be implemented to promote limb function recovery. During the recovery stage, most patients have no obvious pain; systematic rehabilitation treatment is needed. A solid foundation for nerve function recovery can be established by maintaining joint range of motion, preventing adhesive bursitis, strengthening unaffected muscle strength, and using neuromuscular electrical stimulation (NMES) to preserve motor unit activity (1, 30).

Surgery should be considered an alternative for PTS patients who have not responded to or have had poor results from nonsurgical treatment for at least 3 months, although no standard exists at this time (1). Furthermore, we consider surgery if nerve recovery is interrupted or if there is evidence of aberrant (jumping) recovery within 3–6 months. Surgical strategies can draw on principles from the treatment of traumatic nerve repair, although the fundamental natures of the two diseases differ. Nagano (31) advocated nerve decompression as the first option, performing interfascicular neurolysis only, and considered nerve transplantation unnecessary because nerve fibers can recover spontaneously. Concurrently, Pan et al. (14) tried nerve anastomosis or transplantation following the resection of three cases of HGCs during the operation; however, none of these produced the desired outcomes. We believe that the nerve can be completely decompressed if the epineurium is cut open during the procedure and that the majority of the nerve fibers are determined to be in continuity. However, if there is clear evidence of nerve fiber bundle rupture and separation, the lesion should be excised, and nerve end-to-end anastomosis or nerve transplantation should be carried out for repair, as the nerve cannot heal spontaneously when nerve ends are separated. In our study, 2 patients underwent nerve transplantation, and the postoperative muscle strength recovered to Grade 3. However, if there are multiple HGCs and nerve anastomosis cannot be performed, nerve transposition repair of adjacent nerves can be considered, and long-segment nerve transplantation is not recommended (32). However, one patient who underwent nerve transposition of the triceps brachii branch to the axillary nerve achieved only grade 2 muscle strength at the final follow-up, possibly due to severe intrinsic nerve damage. Tendon transfer for functional reconstruction should be considered when muscle strength fails to recover or remains incomplete 18–24 months after onset, fails to meet daily needs, and shows no improvement within 3 months. The two patients who underwent tendon transfer achieved active joint movement against gravity (≥ MRC grade 3), resulting in functional improvement sufficient for daily activities, and both reported satisfaction with the outcome. However, caution is warranted in children, as peripheral nerve injury can lead to central nervous system plasticity imbalances, potentially resulting in postoperative maladaptation and poor outcomes, similar to what is observed in obstetric BP palsy (33, 34). Therefore, it is not recommended to perform the functional reconstruction surgery too early.

Generally, the limb function of most patients can generally meet the needs of daily life after comprehensive treatment, but a small number of patients still experience obvious functional impairment, which may be related to the degree of nerve damage. In this study, 31 of 42 cases achieved effective recovery within two years, which was similar to most reports and verified the effectiveness of our treatment strategy (3, 9, 16, 24). However, the recovery of children in this group was worse than that of adults, which may be related to cortical reorganization or atrophy of relevant brain functional areas due to disuse, as well as children’s limited self-control during the recovery process. Moreover, the body tends to adapt to the impaired state, leading to insufficient spontaneous exercise. Consequently, the development of the affected limb lags behind that of the other limb, creating a vicious cycle of psychological and physiological dysfunction that warrants greater attention. In addition, 21 of the 42 patients still had residual pain, which may be related to muscle compensation caused by weakness of paralyzed muscles; however, effective treatment for this remains lacking (1, 3, 16). Therefore, the expected clinical course and rehabilitation outcomes should be discussed with patients before treatment begins, helping them establish realistic expectations and actively participate in their recovery.

## 5 Conclusion

Our study demonstrates the value of retrospective analysis of clinical data in elucidating the epidemiological and clinical characteristics of PTS in a Chinese cohort. More publicity and education are needed, highlighting the importance of integrating clinical history and physical examination with auxiliary examinations for early diagnosis. Additionally, individualized treatment can improve clinical outcomes, though 50% of patients experienced residual pain and children exhibited poorer prognosis—issues requiring further attention. Notably, our research prioritizes clinical data to reflect the characteristics of PTS, balancing comprehensive characterization with practical clinical implications. The generalizability of our findings is constrained by the small sample size and single-center design. A further limitation is the considerable diagnostic delay, which prevented a systematic evaluation of acute-phase characteristics in PTS. While we endeavored to reconstruct initial symptoms via detailed history-taking, the inherent recall bias is an unavoidable limitation of this retrospective approach. Future large-sample, multi-center prospective studies focusing on Asian populations are warranted to clarify PTS pathogenesis, refine prognostic factors, and optimize treatment protocols.

## Data Availability

The original contributions presented in the study are included in the article/supplementary material, further inquiries can be directed to the corresponding author/s.
